# Potential biomarkers and molecular mechanisms in preeclampsia progression

**DOI:** 10.1515/biol-2022-0053

**Published:** 2022-05-18

**Authors:** Guohua Li, Shijia Huang, Xiaosong Liu, Qiaoling Du

**Affiliations:** Department of Reproductive Immunology, Shanghai First Maternity and Infant Hospital, School of Medicine, Tongji University, Shanghai, 200092, China; Department of Obstetrics, Shanghai First Maternity and Infant Hospital, School of Medicine, Tongji University, Shanghai, 200092, China

**Keywords:** preeclampsia, differentially expressed genes, differentially expressed lncRNAs, ceRNA, trimester

## Abstract

This study aimed to explore potential biomarkers and molecular mechanisms in preeclampsia (PE) progression. Gene expression profiles of GSE147776 and GSE96984 were downloaded, followed by the identification of common differentially expressed genes (co-DEGs) and common differentially expressed lncRNAs (co-DElncRNAs) in PE patients between the two datasets. Key genes were identified using gene set enrichment analysis (GSEA), followed by functional enrichment analyses. Subsequently, the miRNAs of key genes and miRNA-related lncRNAs were predicted, followed by the construction of the lncRNA–miRNA–gene ceRNA network. Furthermore, the key genes associated with different gestational stages were identified. As a result, 192 co-DEGs and 16 co-DElncRNAs were revealed from the two datasets. Based on two outstanding PE-associated pathways, including glaucoma and PE, identified by GSEA, ten key genes, including *IGFBP1*, *CORIN*, and *C3*, were revealed. Key genes, including *IL1A* and *IL1B*, were enriched in the developmental process involved in reproduction. Furthermore, ceRNAs, such as LINC00473-miR-4476-IL1A, LINC00473-miR-1291-IL1B, and NAV2-AS4-miR-6131-REN, were identified. Moreover, REN expression was significantly upregulated in the first- and second-trimester placentae compared to C-section-term placentae. In conclusion, these key genes may serve as novel biomarkers for PE. The detection of REN expression may help in the early prediction of PE and the initiation of prophylactic medical treatment.

## 1 Introduction

Preeclampsia (PE) is a relatively common pregnancy disorder originating in the placenta [1]. The incidence rate of PE, which is directly related to the mortality of women and fetuses, is approximately 5–8% [2]. However, in clinical diagnosis, patients with PE are easily misdiagnosed because the clinical manifestations of PE overlap with other diseases, including hypertension, chronic kidney disease, and primary epilepsy [3]. Therefore, accurate diagnosis and timely intervention are crucial for the clinical treatment of PE.

Factually, gene expression in different molecular patterns is closely associated with PE development [4]. Bioinformatics analysis-based gene profile studies have aided the interpretation of PE progression and can be conducted to identify novel biomarkers of PE [5]. A previous study has showed that certain differentially expressed genes (DEGs), such as cytochrome P450 family 11 subfamily A member 1 (*CYP11A1*), participate in the pathogenesis of PE by inducing excessive autophagy [6]. Moreover, key genes such as glutathione S-transferase omega 1 (*GSTO1*) are considered novel biomarkers for the therapy of PE [7]. Additionally, non-coding RNAs constitute the largest group of RNA transcripts, accounting for 90% of the human genome. Aberrant regulation of specific non-coding RNAs can affect critical mechanisms in PE development, such as immunity, trophoblast proliferation, and trophoblast invasion [8]. MicroRNAs (miRNAs) and long non-coding RNAs (lncRNAs) have been reported to play crucial roles in PE development [9,10,11]. Particularly, lncRNAs may function as competing for endogenous RNAs (ceRNAs) to sponge their target miRNAs, thus mediating the post-transcriptional regulation of target genes by affecting the regulatory function of miRNAs [12]. Identification of lncRNA–miRNA–mRNA ceRNA networks may help elucidate the potential regulatory mechanisms in PE and may aid the discovery of promising biomarkers for PE [13]. Although the relationship between the molecular mechanism and pathological process of PE has been widely studied, its exact pathogenesis and the identity of associated potential diagnostic biomarkers remain uncertain.

**Figure 1:**
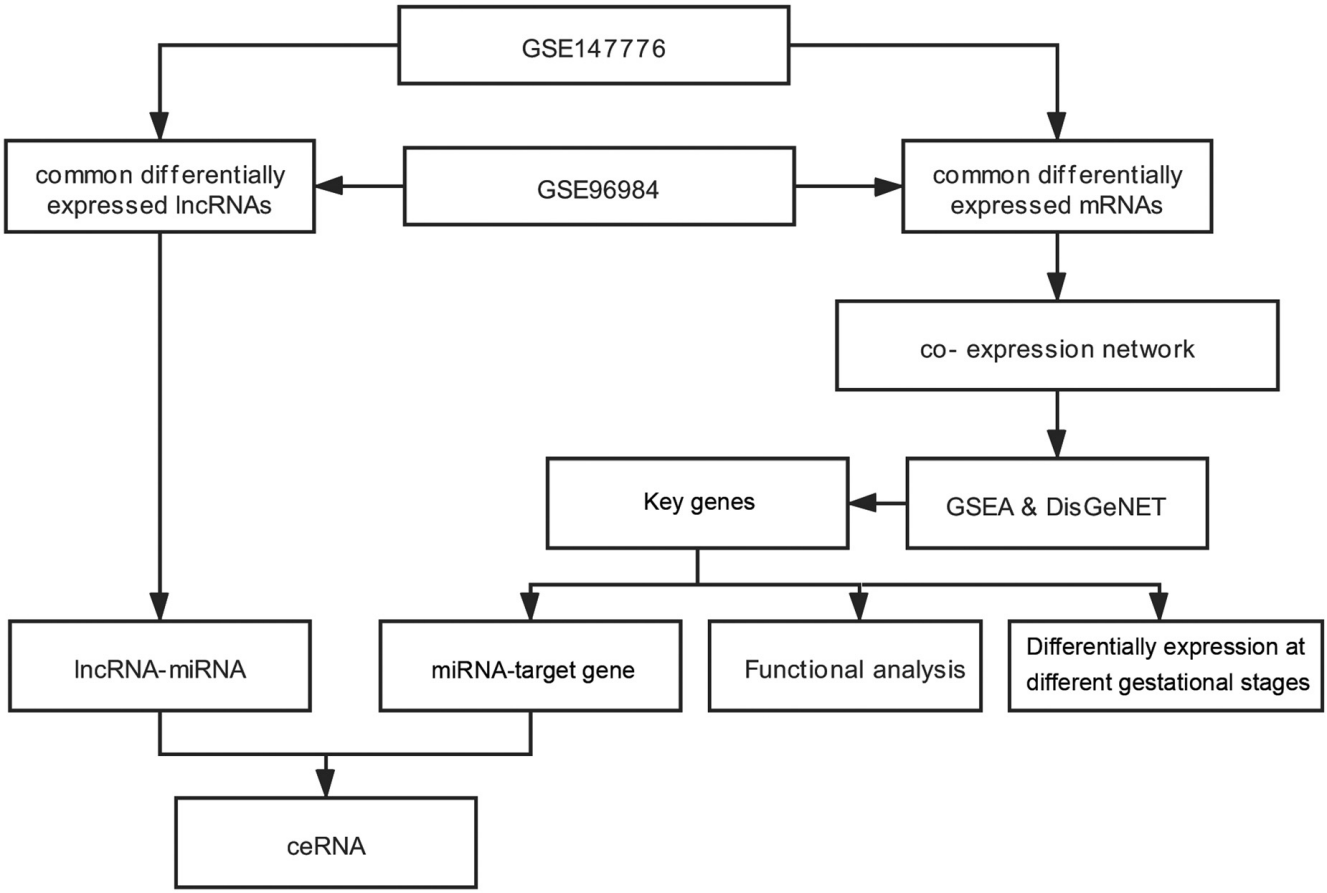
Experimental flow chart of the present study.

A previous placental microarray profiling (GSE147776) study reported by Medina-Bastidas et al. [14] revealed the existence of several DEGs in pregnant women with PE and normal pregnancy (NP). However, the mechanism by which these genes interact with each other and the pathways involved in PE progression remain unknown. In this study, the common DEGs (co-DEGs) in the PE vs NP group between the GSE147776 dataset reported by Medina-Bastidas et al. and another dataset, GSE96984, were investigated, and key gene exploration based on the results of gene set enrichment analysis (GSEA) was performed. The significant functions and pathways enriched by these key genes were identified. Moreover, the common differentially expressed lncRNAs (co-DElncRNAs) in the PE vs NP group between the GSE147776 and GSE96984 datasets were identified. The miRNAs of key genes and miRNA-related lncRNAs were predicted, followed by the construction of the lncRNA–miRNA–gene ceRNA network via integration of the lncRNA–miRNA regulatory relationships and miRNA–target gene pairs. Finally, the key genes associated with different gestational stages were identified to determine the effect of the trimester on PE. The findings of this study may help provide insights into novel biomarkers and molecular mechanisms associated with PE. The flow chart of the present study is shown in Figure 1.

## 2 Materials and methods

### 2.1 Microarray data and preprocessing

The gene expression profile GSE147776 [14] was downloaded from the Gene Expression Omnibus (GEO) database. This dataset included seven tissue samples from patients with intrauterine growth restriction, seven from patients with PE, six from patients with PE and intrauterine growth restriction, and eight from patients with NP. The samples from patients with intrauterine growth restriction were excluded. Thus, seven tissue samples obtained from patients with PE (PE group) and eight from women with NP (NP group) were included in the training set in this analysis. The gene expression profile GSE96984, also downloaded from the GEO database, was used as a validation set in this study. This set contained data on three tissue samples obtained from PE patients (PE group) and four tissue samples obtained from women with normal pregnancy (NP group). Furthermore, using trimester and PE as keywords, only one gene expression dataset, GSE9984, was screened from the GEO database. This dataset included gene expression profiles of three gestational stages (first trimester [6 weeks + 3 days] − [8 weeks + 3 days]), second trimester ([15 weeks + 4 days] − [16 weeks + 3 days]), and C-section-term placentae). There were four placental tissue samples at each gestational stage.

The processed gene expression matrix files in the GSE147776, GSE96984, and GSE9984 datasets were downloaded. The probe IDs were converted to gene symbols based on annotation files downloaded from different platforms. Probes that did not correspond to the gene symbols were discarded. For different probes matched to one gene, the average values of the different probes were used as the final expression values.

### 2.2 DEG investigation and VENN plot analysis

The classic Bayesian method provided by the limma package (version 3.10.3) [15] was used to reveal the DEGs between the PE and NP groups from the GSE147776 and GSE96984 datasets, respectively. Moreover, the DElncRNAs between the PE and NP groups derived from the two datasets were identified using the same method. The threshold values for selecting DEGs and DElncRNAs were set as *P-*value < 0.05 and |log fold change (FC)| > 1.5. VENN plot analyses were subsequently performed to identify the co-DEGs and co-DElncRNAs exhibiting the same up- or downregulation of expression in the two datasets. Co-DEGs and co-DElncRNAs were included in the subsequent analysis.

### 2.3 Differential co-expression correlation analysis

The differential co-expression correlation of co-DEGs was revealed using the Diffcorr package (version: 0.4.1, https://CRAN.R-project.org/package = DiffCorr) [16] in R software. Briefly, the Pearson correlation coefficient of each co-DEG was calculated, followed by a correlation test. The co-DEGs with |*r* (*r*_1_ − *r*_2_)| > 1 (with *r*_1_ being the correlation coefficient of two genes in the NP group and *r*_2_ being the correlation coefficient of two genes in the PE group) were considered as differentially co-expressed genes. The co-expression relations in both NP and PE groups were further explored with |*r*| > 0.8, and *P* < 0.05. Finally, the co-expression network in the two groups was visualized using the Cytoscape software (version 3.4.0) [17], followed by the conduction of network topology (closeness centrality and degree centrality) analysis.

### 2.4 GSEA based on co-DEGs and information in the disease database

GSEA is a commonly used method for analyzing gene sets generated via the conduction of genome-wide experiments [18]. It can be used to identify significant differences in gene expression between the two different biological states [19]. Based on the gene–disease associations obtained from the DisGeNET database [20], GSEA was performed in an orderly manner (according to the descending order of the log FC) for all genes in GSE147776 using the clusterProfiler package (version: 3.16.0, http://bioconductor.org/packages/release/bioc/html/clusterProfiler.html) [21]. The Benjamini & Hochberg-adjusted *P* < 0.05 was considered as the threshold value for conducting analysis in the present study.

### 2.5 Investigation of key genes of PE

The key genes of PE were investigated based on the results of GSEA and co-expression correlation analysis. Briefly, the genes that were reported in the database of PE-associated disease (revealed by GSEA), along with the exhibition of co-expression in co-expression correlation analysis, were considered key PE genes. The differential expression of these key genes in different clinical phenotypes was also analyzed. Finally, the results were visualized and illustrated using a box diagram.

### 2.6 Enrichment analysis of key genes

Gene Ontology-Biological Process (GO-BP) [22] and Kyoto Encyclopedia of Genes and Genomes (KEGG) pathway [23] enrichment analyses of key genes were performed using the Metascape software (parameter: minimum overlap = 3; minimum enrichment = 1.5) [24]. *P* < 0.05 was considered the cutoff value to obtain significant enrichment results. Moreover, clustering analysis was conducted according to the similarity of genes enriched in each term (similarity >0.3). The most statistically significant term (minimum *P*-value) in each cluster was selected to define the cluster. Finally, the network was constructed using the Cytoscape software (version 3.4.0) [17].

### 2.7 Prediction of miRNAs of key genes and construction of the lncRNA–miRNA–gene ceRNA network

The miRNAs of key genes were predicted using miRWalk 3.0 (http://mirwalk.umm.uni-heidelberg.de/) [25]. The obtained miRNA–target gene pairs were further subjected to screening using miRDB (http://mirdb.org/) [26]. The miRNA–target gene pairs with scores > 0.95 were selected for network construction. Additionally, the miRNA-related lncRNAs were predicted using the Prediction Module of DIANA-LncBase v.2 (http://carolina.imis.athena-innovation.gr/diana_tools/web/index.php?r=lncbasev2%2Findex) [27]. The lncRNA–miRNA regulatory relationships with scores > 0.7 were then obtained. The lncRNA–miRNA regulatory relationships containing co-DElncRNAs were further screened. By integrating the lncRNA–miRNA regulatory relationships and miRNA–target gene pairs, the lncRNA–miRNA–gene ceRNA network was constructed.

### 2.8 Identification of key genes associated with different gestational stages

To determine the effect of the trimester (different gestational ages) on PE, based on the gene expression data of three gestational stages ([6 weeks + 3 days] − [8 weeks + 3 days], second trimester [15 weeks + 4 days] − [16 weeks + 3 days], and C-section-term placentae) in GSE9984 datasets, the expression of key genes between any two gestational stages was compared using the *t*-test in R.

## 3 Results

### 3.1 DEG and co-DEG investigation

**Figure 2:**
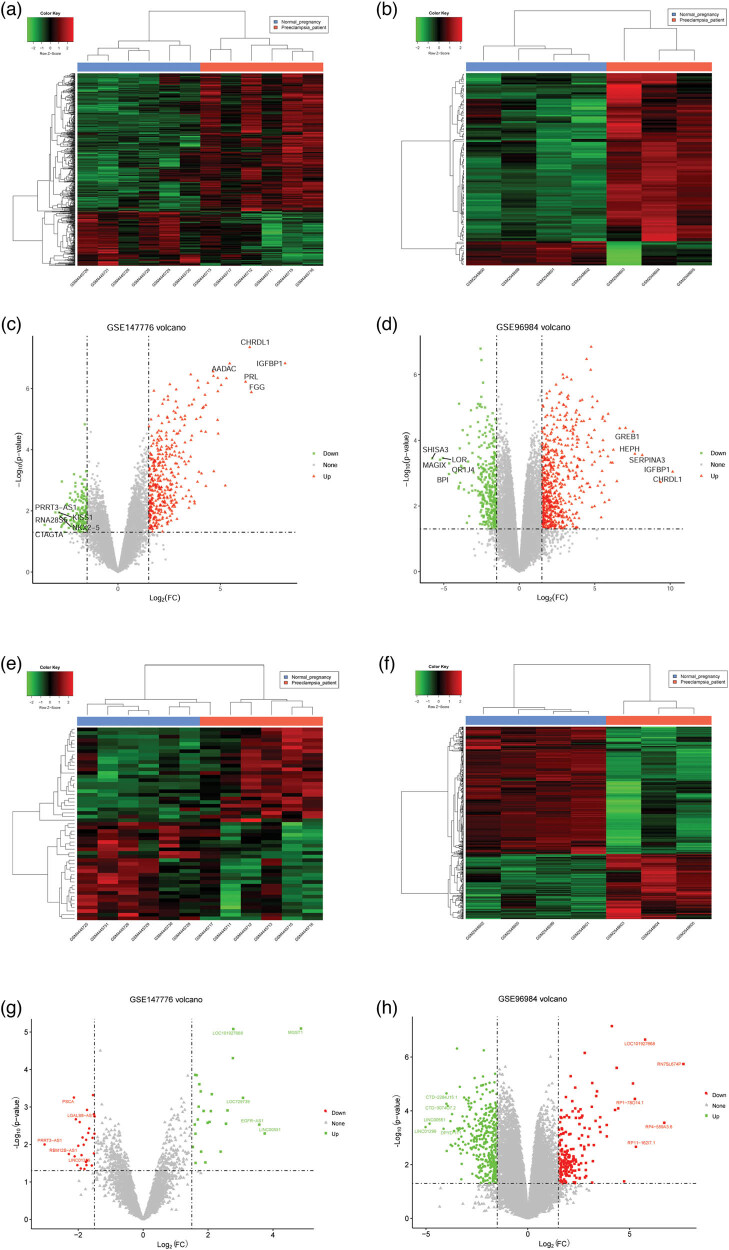
Results of DEGs and differentially expressed lncRNAs (DElncRNAs) obtained during investigation. (a) The heat map illustrating DEGs between the PE and NP groups in GSE147776; (b) the heat map illustrating DEGs between PE group and NP group in GSE96984. (c) The volcano plot illustrating the expression of genes in GSE147776; (d) the volcano plot illustrating the expression of genes in GSE96984; the green node represents downregulated genes, while the red node represents the upregulated genes; (e) the heat map illustrating DElncRNAs between the PE and NP groups in GSE147776; (f) the heat map illustrating DElncRNAs between the PE and NP groups in GSE96984. (g) The volcano plot illustrating the expression of DElncRNAs in GSE147776; (h) the volcano plot illustrating the expression of DElncRNAs in GSE96984; the green node represents downregulated lncRNAs, while the red node represents upregulated lncRNAs.

**Figure A1:**
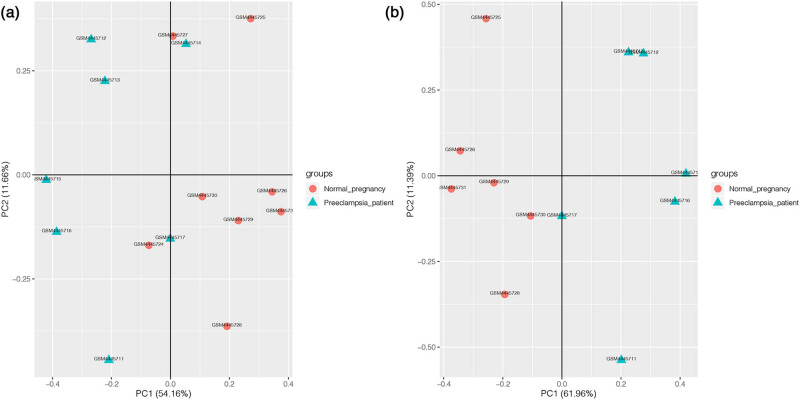
The principal component analysis (PCA) results for GSE147776. (a) Results of PCA before abnormal sample exclusion; (b) results of PCA after abnormal sample exclusion.

A total of 23,825 genes and 4,720 lncRNAs, and 18,826 genes and 19,741 lncRNAs, were annotated in GSE147776 and GSE96984, respectively. Based on principal component analysis (PCA) results, three samples in GSE147776, GSM4445727, GSM4445714, and GSM4445724, were excluded due to abnormal expression observed between the PE and NP groups (Figure A1). All samples in GSE96984 were included for DEG analysis as no abnormal samples were observed using PCA. A total of 554 DEGs (154 downregulated and 390 upregulated) and 968 DEGs (363 downregulated and 605 upregulated) between the PE and NP groups were obtained in GSE147776 and GSE96984, respectively. The heatmap of the clustering analysis conducted for DEGs indicated that the samples in the PE and NP groups could be clearly distinguished in GSE147776 (Figure 2a) and GSE96984 (Figure 2b). The volcano plot depicts the DEGs in GSE147776 (Figure 2c) and GSE96984 (Figure 2d). Additionally, 53 DElncRNAs (27 downregulated and 26 upregulated) and 571 DElncRNAs (378 downregulated and 193 upregulated) were identified between the PE and NP groups in GSE147776 and GSE96984, respectively. Heatmap and volcano plots for DElncRNAs in the two datasets are illustrated in Figure 2e–h.

**Figure 3:**
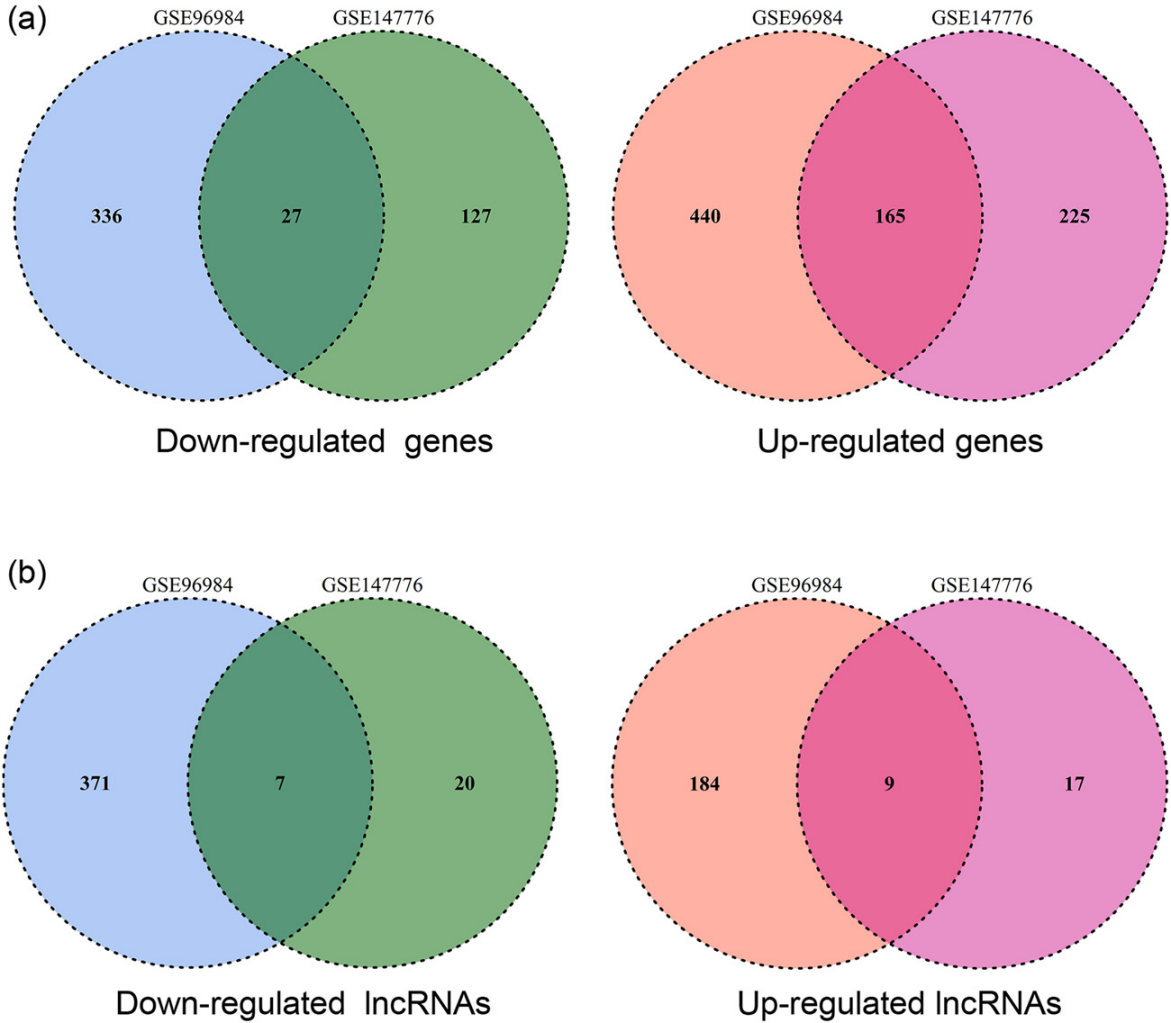
VENN plot illustrating common DEGs (co-DEGs) and common DElncRNAs (co-DElncRNAs) between GSE96984 and GSE147776 datasets. (a) The common downregulated and upregulated genes observed between the two datasets. (b) The common downregulated and upregulated lncRNAs observed between the two datasets.

Furthermore, the co-DEGs and co-DElncRNAs between the GSE147776 and GSE96984 datasets were explored via VENN plot analysis. The results showed that there were 192 co-DEGs (27 downregulated and 165 upregulated) (Figure 3a) and 16 co-DElncRNAs (7 downregulated and 9 upregulated) (Figure 3b) between the two datasets.

### 3.2 Differential co-expression correlation and co-expression network analysis

**Figure 4:**
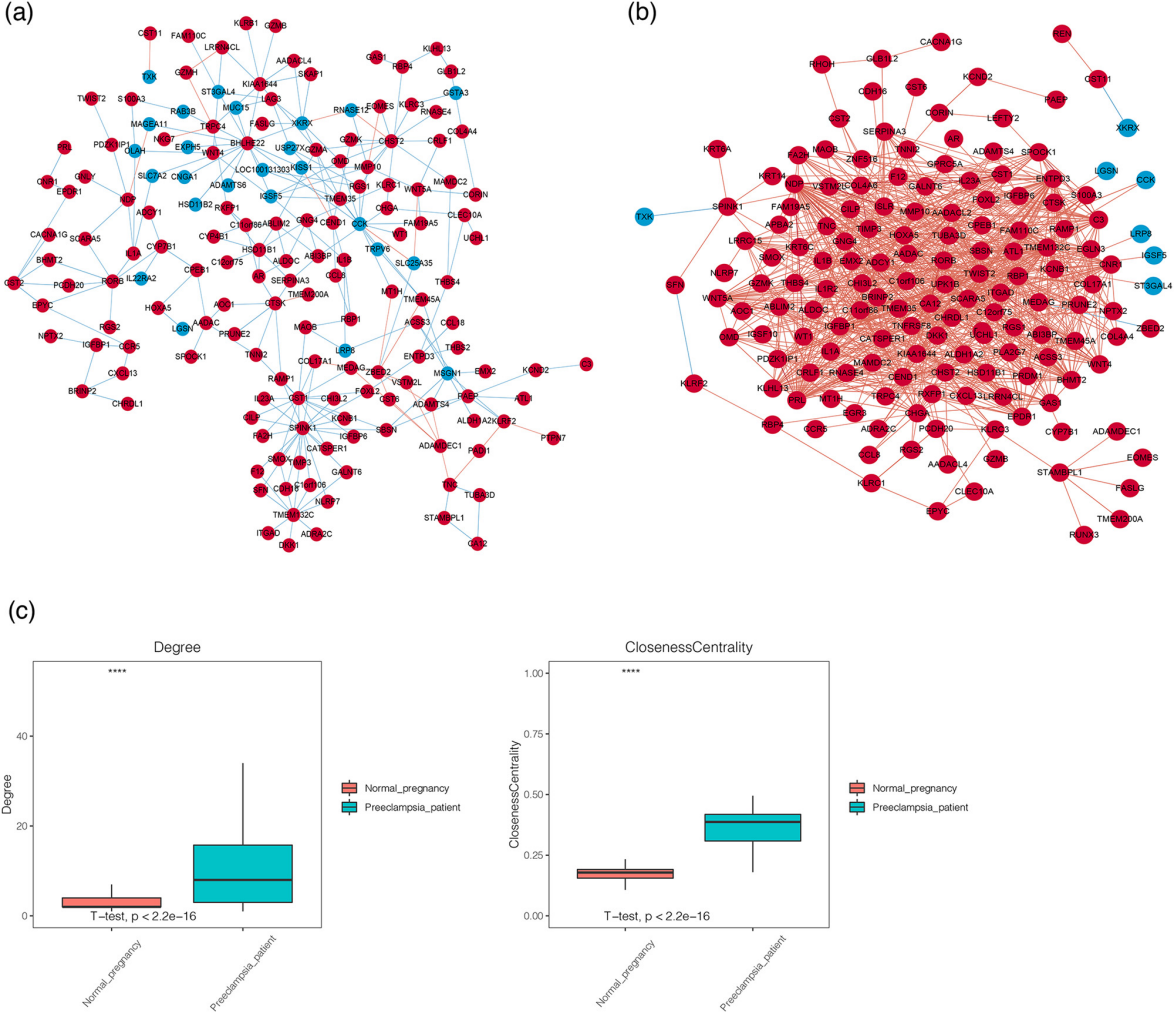
Co-expression networks constructed by co-DEGs. (a) The co-expression network constructed by co-DEGs in the NP group. (b) The co-expression network constructed by co-DEGs in the PE group. The red node represents upregulated genes; the blue node represents downregulated genes; the blue line represents negative co-expression; the red line represents positive co-expression. (c) The degree centrality and closeness centrality between NP and PE groups. The red block represents the NP group, while the blue block represents the PE group.

A total of 1,365 differential co-expression correlations were further investigated based on 192 co-DEGs using the Diffcorr package in R software. The co-expression networks in both the NP and PE groups were constructed using 1,365 differentially co-expressed elements. The results showed that there were 166 co-DEGs and 253 correlations in the co-expression network of the NP group (Figure 4a). A total of 158 co-DEGs and 891 correlations were observed in the co-expression network of the PE group (Figure 4b). The topological property analysis of the two networks showed that, compared with the NP group, the degree centrality and closeness centrality of the PE group were significantly increased (Figure 4c, *P* < 0.05), which further indicated that co-expression of these genes might contribute to the occurrence of diseases.

### 3.3 Investigation of key genes based on GSEA

**Figure 5:**
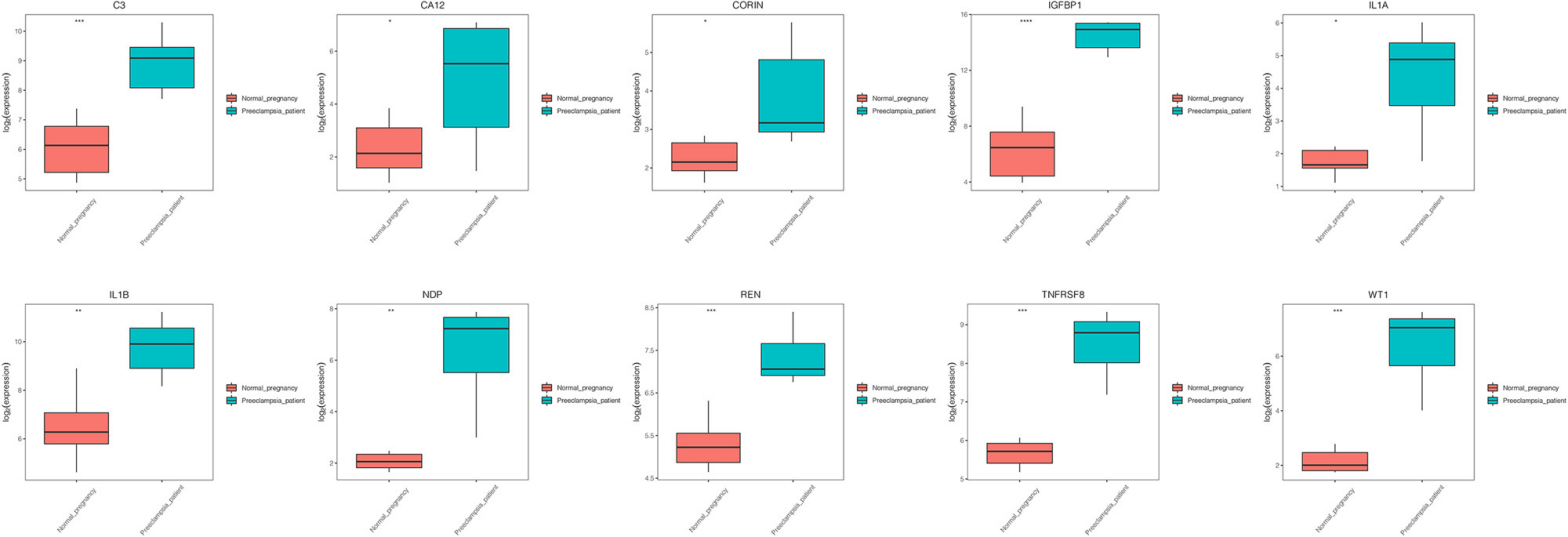
The expression of ten key genes in the NP and PE groups. The red block represents the NP group, while the blue block represents the PE group.

**Figure A2:**
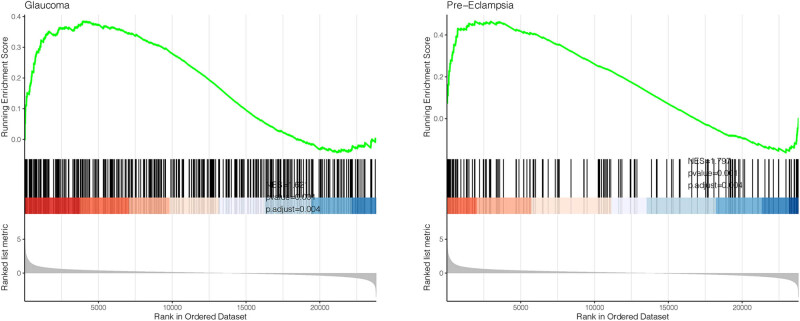
The results of gene set enrichment analysis performed for two significantly enriched pathways, including glaucoma and preeclampsia.

Based on the DisGeNET database and logFC-sequenced genes in the GSE147776 dataset, a total of 14 PE-related diseases, such as maternal hypertension, glaucoma, and PE, were revealed (Table A1). GSEA analysis showed that co-DEGs were mainly enriched in glaucoma (C0017601) and PE (C0032914) (Table A1 and Figure A2). During the analysis of enrichment of co-DEGs in the two pathways, TNF receptor superfamily member 8 (*TNFRSF8*), norrin cystine knot growth factor NDP (*NDP*), complement C3 (*C3*), WT1, insulin-like growth factor binding protein 1 (*IGFBP1*), interleukin-1A (*IL1A*), corin/serine peptidase (*CORIN*), interleukin-1B (*IL1B*), renin (*REN*), and carbonic anhydrase 12 (*CA12*) were identified as key genes of PE, as the expression levels of the indicated genes were significantly upregulated in the PE group (Figure 5).

### 3.4 GO and KEGG enrichment analyses of key genes

GO-BP and KEGG analyses were performed for the indicated ten key genes. The results showed that the genes were mainly assembled in 43 GO-BP functions and 4 pathways. Clustering analysis according to the observation of similarity of key genes enriched in each term was subsequently performed. The results showed that a total of five clusters, including positive regulation of vascular endothelial growth factor production (GO: 0010575; genes: *C3*, *IL1A*, and *IL1B*), developmental process involved in reproduction (GO: 0003006; genes: *IL1A*, *IL1B*, *NDP*, *REN*, and *WT1*), endocrine process (GO: 0050886; genes: *IL1B*, *REN*, and *CORIN*), response to organophosphorus (GO: 0046683; genes: *IL1B*, *REN*, and *WT1*), and visual system development (GO: 0150063; genes: *C3*, *NDP*, and *WT1*) were explored.

### 3.5 Construction of the lncRNA–miRNA–gene ceRNA network

**Figure 6:**
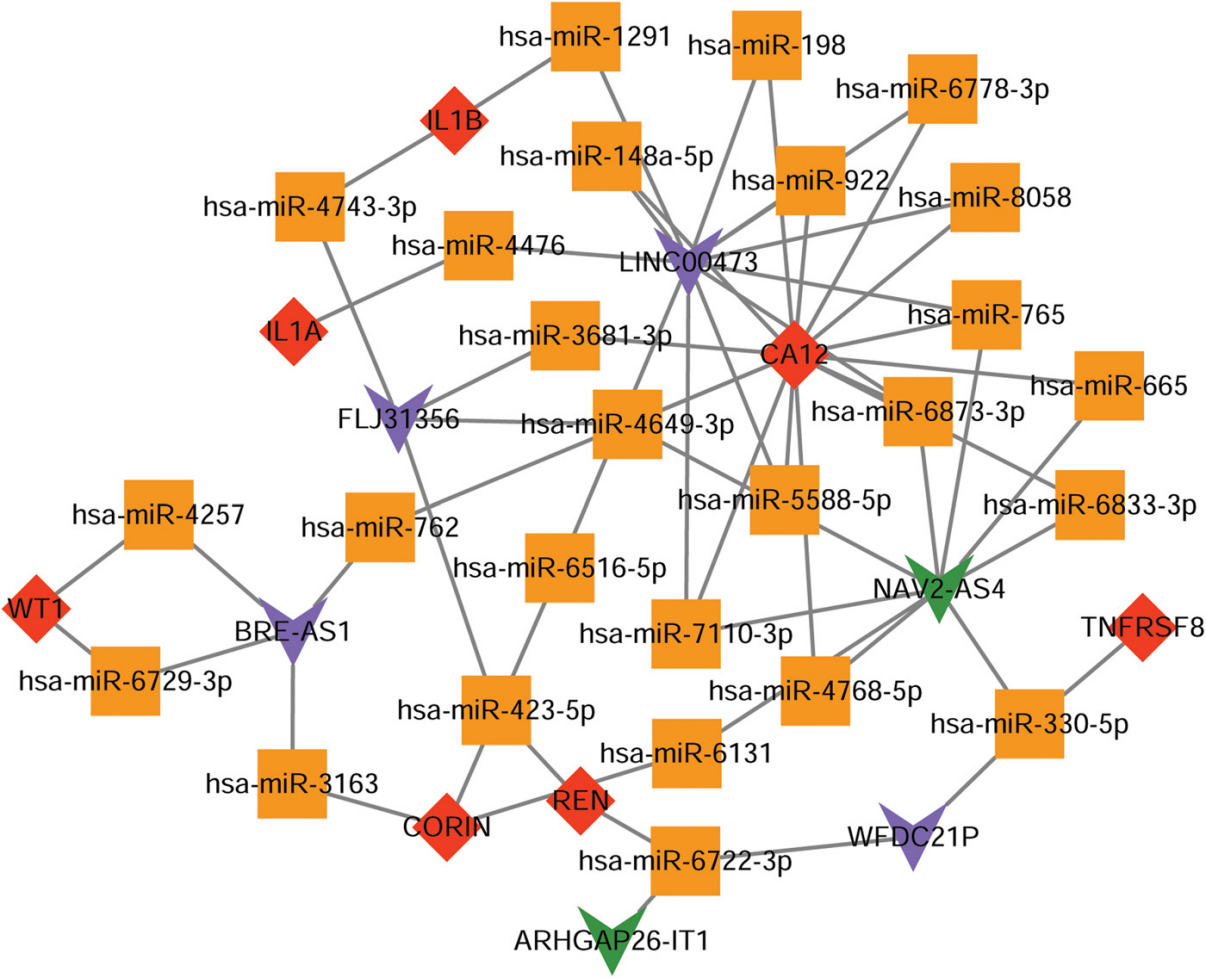
The constructed lncRNA–miRNA–gene ceRNA network. The red diamond node represents the upregulated gene, the orange square node represents miRNA, the purple inverted triangle node represents the upregulated lncRNA, and the green inverted triangle node represents the downregulated lncRNA.

**Figure A3:**
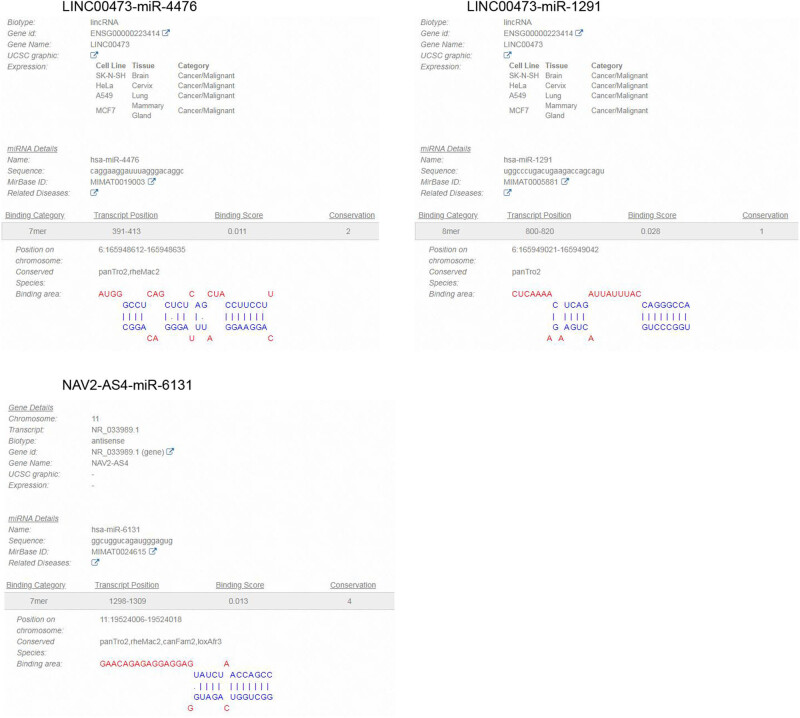
The complementary sequences of LINC00473-miR-4476, LINC00473-miR-1291, and NAV2-AS4-miR-6131.

A total of 99 miRNA–target gene pairs were obtained, including 97 miRNAs and 8 key genes. Data on 6 co-DElncRNAs and 26 miRNAs were obtained by further predicting lncRNA–miRNA regulatory relationships. The lncRNA–miRNA–gene ceRNA network was constructed by integrating the lncRNA–miRNA regulatory relationships and miRNA–target gene pairs, including 58 interactions, 26 miRNAs, 6 lncRNAs, and 7 key genes (Figure 6). Notably, among the six lncRNAs, LINC00473 could target most miRNAs, followed by NAV2-AS4. LINC00473-miR-4476-IL1A, LINC00473-miR-1291-IL1B, and NAV2-AS4-miR-6131-REN were identified as key ceRNAs. The complementary sequences of LINC00473-miR-4476, LINC00473-miR-1291, and NAV2-AS4-miR-6131 are shown in Figure A3.

### 3.6 Identification of key genes associated with different gestational stages

**Figure 7:**
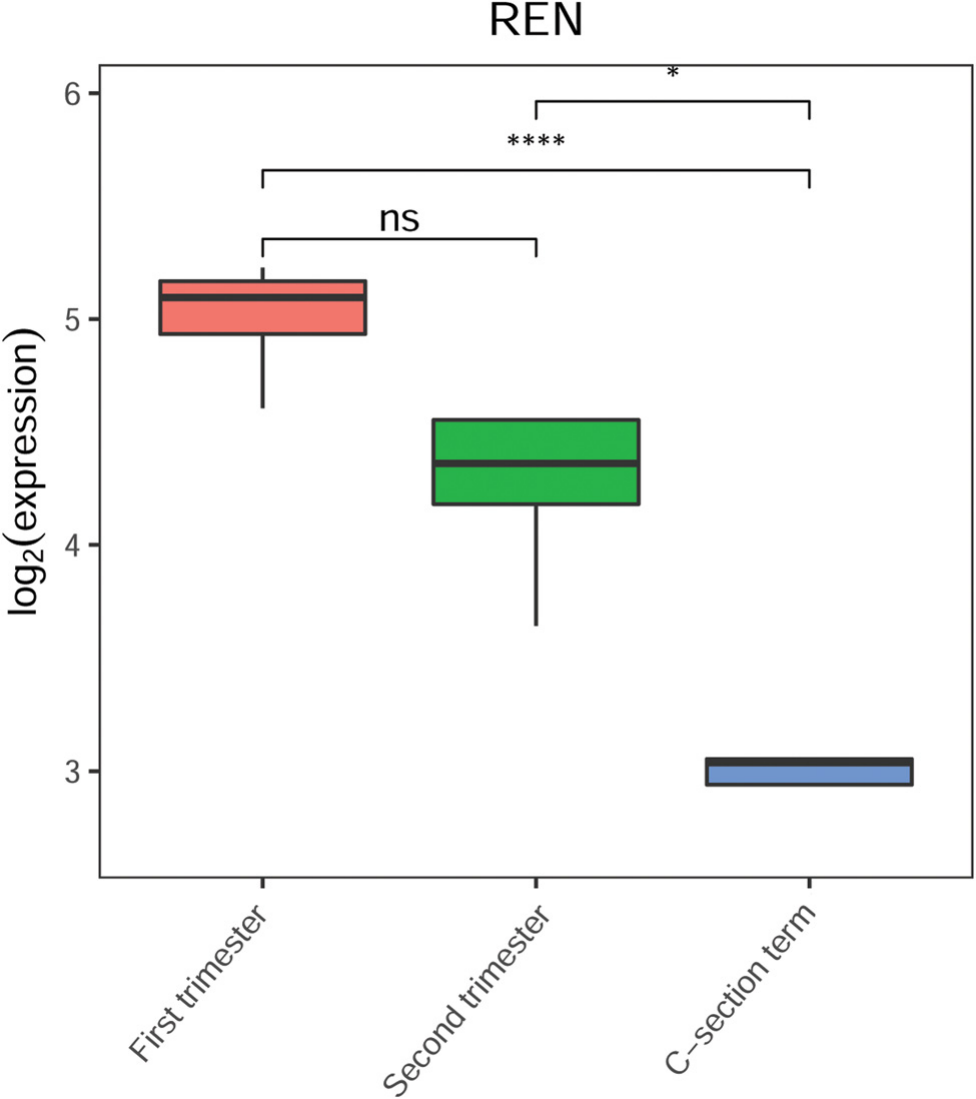
The expression of REN at different gestational stages. The red block represents the first-trimester group, the green block represents the second-trimester group, and the blue block represents the C-section term group.

The expression of key genes at different gestational stages was analyzed based on the gene expression data of three gestational stages in the GSE9984 datasets. The results showed that only REN was differentially expressed among the different gestational stages. Notably, the expression of REN gradually decreased with an increase in gestation time. REN expression in the first- and second-trimester placentae was significantly higher than that in the C-section-term placentae (Figure 7). These data indicate that the detection of REN expression may be useful for the early prediction of PE.

## 4 Discussion

Although PE is a pregnancy-associated complication exhibiting considerable consequences in women, the biomarkers and associated molecular mechanisms underlying PE progression remain unclear. The present bioinformatics study revealed two remarkable pathways (glaucoma and PE) using GSEA based on 14 PE-related diseases and co-DEGs. A total of ten co-DEGs, including *IGFBP1*, *CORIN C3*, *IL1A*, *IL1B*, and *REN*, were identified as key genes. Moreover, functional cluster analysis results showed that these key genes, including *IL1A* and *IL1B*, were mainly involved in functions involved in reproduction, such as developmental processes. Furthermore, LINC00473-miR-4476-IL1A, LINC00473-miR-1291-IL1B, and NAV2-AS4-miR-6131-REN were identified as key ceRNAs in the lncRNA–miRNA–gene ceRNA network. Additionally, we found that REN expression was significantly upregulated in first- and second-trimester placentae compared to that in the C-section-term placentae, indicating that the detection of REN expression might be useful for early prediction of PE.

As PE is a common vascular disease associated with pregnancy with a known genetic predisposition, the abnormal expression of certain genes during this process contributes to the progression of PE [28]. *IGFBP1* plays essential role in embryogenesis and carcinogenesis [29]. Under normal circumstances of clinical observation, the expression of *IGFBP1* in pregnant women is reportedly higher than that in non-pregnant women [30]. However, low expression of *IGFBP1* has been observed in the maternal blood samples derived from PE patients compared to healthy women [31]. In contrast, an investigation based on the alteration of serum IGFBP-1 concentration in women with different stages of eclampsia showed that IGFBP-1 levels in the PE group were significantly higher than those in the control group, which was related to the severity of PE [32]. It has been proven that the expression of the *IGFBP1* gene is abnormal before PE development and can be clinically detected due to abnormalities in the trophoblastic invasion, thereby highlighting potential applicability as a marker for the identification of PE [33]. Moreover, CORIN encodes an atrial natriuretic peptide-converting enzyme [34]. The CORIN-mediated atrial natriuretic peptide in the uterus under pregnancy conditions promotes spiral artery remodeling and trophoblast invasion [35]. A previous study showed that the differential expression of the *CORIN* gene induced by two mutations (K317E and S472g) could be observed in PE patients, which might contribute to the development of PE [36]. The potential consideration of *CORIN* in PE clinical treatment as a new biomarker has been investigated in a previous study [37]. Furthermore, C3 is a complement component with the highest level in serum and plays an important role in both the classical and bypass activation pathways [38]. A previous genetic case–control study based on 960 PE patients has revealed that C3 plays a potential role in the complement system in the pathogenesis of PE, and may be considered a prognostic and therapeutic biomarker of preeclamptic women [39]. It has been proven that the gene mutation of C3 (such as C3F) is associated with susceptibility to PE [40]. In the present study, *IGFBP-1*, *CORIN*, and *C3* were identified as three key genes with upregulated expression between the PE and NP groups. Meanwhile, GSEA showed that all three genes were significantly enriched in pathways such as those in PE. Thus, we speculated that expression levels of *IGFBP-1*, *CORIN*, and *C3* were upregulated during the development of PE, and the three genes might therefore be considered novel biomarkers for PE.

The occurrence and development of PE is closely associated with the functions of the human reproductive system [41]. The occurrence of PE represents a reproductive disadvantage unique to humans compared to other mammals [42]. Reproduction and associated factors, such as neutrophil extracellular traps, play a vital role in the development of pregnancy-related disorders in PE [43]. A previous study has indicated that the differential expression of certain genes in the reproduction pathway influences the risk of PE [44]. The interleukin-1 (IL1) family is a group of 11 cytokines (including IL1A and IL1B) that form a complex network of proinflammatory cytokines that initiate and control inflammatory responses through integrin expression on leukocytes and endothelial cells [45]. IL-1 system components, including IL1A and IL1B, have been shown to exhibit several sites of synthesis in the reproductive system, including the ovary [46]. In fact, the genetic variations of the IL1 family member IL1A are related to PE risk in the Chinese Han population [47]. A previous study showed that IL1A could be considered a therapeutic tool for monitoring PE patients because it leads to a proinflammatory cascade and results in high levels of circulating cytokines involved in PE progression [48]. Moreover, univariate analysis results suggest that the inflammatory process-related gene *IL1B* is involved in the host response of PE [49]. In this study, the IL-1 family members *IL1A* and *IL1B* were not only revealed as upregulated key genes in the PE and NP groups but also found to be significantly assembled during the developmental process involved in reproduction. Thus, we speculated that *IL1A* and *IL1B* might participate in the progression of PE by affecting developmental processes involved in reproduction. Additionally, LINC00473-miR-4476-IL1A and LINC00473-miR-1291-IL1B were identified as key ceRNAs in the lncRNA–miRNA–gene ceRNA network. LINC00473 reportedly contributes to PE development and may serve as a potential biomarker for PE [50]. Although miR-4476 and miR-1291 have been revealed to play a role in the development of various cancers [51,52,53], their possible role in PE development has not been reported and warrants further study. Our results suggest that these ceRNAs may be key mechanisms mediating PE development and may provide novel biomarkers for PE.

Notably, the first-trimester prediction of PE has important clinical implications, as it allows clinicians to focus on high-risk populations and aids the development of prophylactic medical treatment strategies. Herein, we found that the expression of REN in first and second-trimester placentae was significantly higher than that in the C-section-term placentae. Previous studies have confirmed that REN is associated with PE, and REN gene polymorphisms play a crucial role in PE development [54,55]. Moreover, NAV2-AS4-miR-6131-REN was identified as a key ceRNA in our study. However, the roles of NAV2-AS4 and miR-6131 in PE have not been elucidated. Considering the potential role of REN in PE, we speculate that detection of REN expression may demonstrate utility for early prediction of PE, and NAV2-AS4 and miR-6131 may also be involved in PE via regulation of REN expression.

However, there were some limitations in the present study. First, the sample size was small. Second, there was a lack of clinical verification. Finally, whether the selected lncRNA–miRNA–mRNA was dysregulated under hypoxic conditions was not explored in trophoblast cells *in vitro.* Thus, further experimental verification based on a larger sample size is imperative.

In conclusion, expression levels of key genes, such as *IGFBP1*, *CORIN*, *C3*, *IL1A*, *IL1B*, and *REN*, were upregulated during the development of PE, and these genes could therefore be considered novel biomarkers for PE. Moreover, *IL1A* and *IL1B* may participate in the progression of PE via developmental processes involved in reproduction. Furthermore, key ceRNAs, such as LINC00473-miR-4476-IL1A, LINC00473-miR-1291-IL1B, and NAV2-AS4-miR-6131-REN, may also contribute to PE development. Detection of REN expression may be useful for the early prediction of PE and may aid the initiation of prophylactic medical treatment.

## Appendix

**Table A1:** The enrichment result using GSEA

**ID**	**Description**	SetSize	EnrichmentScore	P. adjust	CoreEnrichment
C0017601	Glaucoma	357	0.384723753	0.00401984	NDP/WT1/TTR/IL1B/CD69/CA12/IL1A/GSTT1/ROM1/NOS2/HLA-DQB1/IFT172/PTPN22/FN1/PTGER3/DAPK2/MMP3/TUB/GJB3/NOD2/TNF/SAA2/CA2/NTRK1/ANTXR1/PITX2/HLA-DRB1/TTC8/AGT/SFRP1/DDX58/ACE2/MGP/OPTN/PLK4/ARL6/COCH/NCAM1/VEGFA/EDNRB/VCAN/TMEM98/MMP1/GSTM1/PUS1/SALL1/BEST1/ABCA4/FAT4/TMEM182/COL4A1/FAS/CA4/GSTM3/IFIH1/NPPA/ADAMTS17/EDNRA/TLR4/BCOR/RPGR/ADAMTS10/WDR36/LTBP2/CDKN2A/CLU/IDUA/SPATA7/CTGF/ADRB2/PIK3R1/SPARC/IL6/TMCO1/NFKB1/PRPH2/TDRD7/CYP1B1/C1QA/BBS2/SLC4A4/TGFB2/GSTM2/RPGRIP1
C0032914	Pre-Eclampsia	146	0.465393454	0.00401984	IGFBP1/TAC3/C3/TNFRSF8/CXCL10/CD40LG/IL15/IFNG/REN/SERPIND1/NOS2/CORIN/CCL5/MMP9/SERPINE1/SELL/TAP1/HDC/NOD2/TNF/ADA/BDKRB2/VCAM1/S100B/AGT/PYGM/VEGFA/NOV/IL18/LGALS1/TAP2/PRCP/CYR61/HP/HLA-G

**Notes:** GSEA, gene set enrichment analysis; EnrichmentScore: positive values indicate up-regulated genes and negative values indicate down regulated genes; Core Enrichment: the core genes with the most contribution to the enrichment score.
